# Comparison the cost-efficacy of furazolidone-based versus clarithromycin-based quadruple therapy in initial treatment of *Helicobacter pylori* infection in a variable clarithromycin drug-resistant region, a single-center, prospective, randomized, open-label study

**DOI:** 10.1097/MD.0000000000014408

**Published:** 2019-02-08

**Authors:** Dong-Min Yi, Tao-Tao Yang, Shuai-Heng Chao, Ya-Xin Li, Ying-Lei Zhou, Hai-Hui Zhang, Ling Lan, Yu-Wei Zhang, Xue-Mei Wang, Yan-Rui Zhang, Jian Li, Song-Ze Ding

**Affiliations:** a Department of Gastroenterology and Hepatology; b Department of Experimental Medicine; c Department of Genetic Medicine; d Department of Traditional Chinese Medicine, People's Hospital of Zhengzhou University, Jin Shui District, Zhengzhou, Henan, China.

**Keywords:** amoxicillin, clarithromycin, cost-effectiveness, eradication, furazolidone, *Helicobacter pylori*, quadruple therapy

## Abstract

*Helicobacter pylori* (Hp) drug resistant rate to clarithromycin (CLA) has increased to 20% to 50%, which cause concerns regarding its effectiveness in eradicating Hp, we aim to evaluate the cost-effectiveness of CLA-based versus furazolidone (FZD)-based quadruple therapy, and assess factors that affect anti-Hp efficacy.

One hundred eighty-five patients were enrolled in this single-center, prospective, randomized, open-label study. In FZD group, 92 patients were treated with FZD plus esomeprazole, bismuth potassium citrate, and amoxicillin for 14 days. In CLA group, 93 patients were treated with the same regimen except FZD was replaced by CLA. Patients were tested 4 weeks post-treatment to confirm eradication.

Of the 185 enrolled patients, 180 completed the study. On intention-to-treat analysis, Hp eradication rates in FZD and CLA groups were 90.22% and 86.02% (*P* = .378); in per-protocol analysis, their eradication rates were 93.26% and 87.91%, respectively (*P* = .220). Overall incidence of total side effects in FZD and CLA groups was 19.57% and 13.98%, and their severe side effects were 3.26% and 2.15%, respectively (*P* > .05). Cost-effectiveness ratios of FZD and CLA groups were 0.75 and 1.02, and incremental cost-effectiveness ratio of FZD group over CLA group was −3.62. Eradication failures were not associated with factors including gender, age, body mass index, smoking, alcohol consumption, educational level, and urban–rural distribution in this observation (*P* > .05).

Despite increasing drug resistance to CLA, Hp eradication rates in FZD and CLA groups have no significant difference at present; as FZD-based quadruple therapy is more cost-effective, we recommend this regimen be a first-line choice for Hp eradication.

## 1 Introduction

Drug-resistance rates of *Helicobacter pylori* (Hp) to clarithromycin (CLA), metronidazole, and levofloxacin have been increasing over the past decades in many regions of the world and in China. Accordingly, the eradication regimens containing these medicines usually result in poor treatment effectiveness.^[1–6]^ Reports have indicated that the primary drug-resistance rates of Hp to CLA range from 20% to 50%, to metronidazole are 40% to 70%, to levofloxacin at 20% to 50%, but to amoxicillin (AMX) only at 0% to 5%, and to furazolidone (FZD) at 0% to 1% in Chinese population.^[1,6]^ Therefore, selection of proper treatment regimen with high eradication rates and fewer drug-resistant incidences is of critically important for Hp eradication. Quadruple therapies with proton pump inhibitor (PPI), bismuth, and combination of 2 antibiotics have recently been recommended as one of the preferred options for anti-Hp therapy by several national guidelines.^[1,2]^

FZD is a nitrofuran antibiotic, which has been historically used in treatment of peptic ulcers, and has shown high potency and safety for Hp eradication especially when used together with bismuth compounds.^[7]^ A meta-analysis on efficacy and safety of FZD containing regimen has indicated that FZD-regimen is superior to other antibiotic-containing therapies, such as metronidazole-containing therapy, and the eradication rate could reach 92.9% (95% confidence interval [CI]: 90.7–95.1) by per-protocol (PP) analysis.^[8]^ Liang et al^[9]^ use 14-day quadruple regimen that contains lansoprazole, bismuth potassium citrate, AMX, and FZD to treat patient and achieve eradication rates of 99.0% in PP analysis, and 95.2% in intention-to-treat (ITT) analysis, respectively.

Studies have also reported that FZD-based quadruple regimens at low FZD dose (100 mg bid) fail to yield acceptable eradication rates^[10]^; at higher dose (200 mg bid), the eradication rate is significantly increased, but incidences of side effect are also occurred more frequent, mostly due to gastrointestinal discomfort, which have affected the use of FZD in Hp eradication.^[8,10,11]^ Among the commonly used drugs to eradicate Hp, AMX provides lower drug-resistance rate, and its secondary resistance rate is also very low.^[1,12]^

However, comparison the cost and efficacy of bismuth quadruple regimen combined with FZD, AMX, and/or CLA for eradication of Hp is lacking clinical data in this region which has variable Hp infection rates, and it is not clear if FZD–AMX combination is superior to present commonly used bismuth–CLA-based quadruple therapy. In this study, we compare the efficacy, safety, cost, and compliance of FZD-based quadruple regimen with routine CLA-based quadruple therapy in initial treatment for Hp-infected patients, and identify factors that affect eradication efficacy in order to obtain an optimal approach for clinical practice.

The results indicate that FZD-based quadruple therapy including esomeprazole, FZD, bismuth potassium citrate, and AMX for 14 days provides satisfactory results for Hp eradication; despite increasing CLA-resistance incidences, CLA-based treatment still achieve acceptable effects in this region, although it is not as cost-effective as FZD-based regimen. These results provide insights and options for choosing optimal regimen in clinical practice during treating Hp infection-related upper gastrointestinal disorders.

## 2 Materials and methods

### 2.1 Patients

This single-center, prospective, randomized control open-label study was conducted at People's Hospital of Zhengzhou University, in Zhengzhou, Henan province, China. Henan province is the largest province in China with a population near 100 million, and social-economic conditions vary greatly between urban and rural areas. From October 2015 to May 2017, a total of 185 patients were enrolled from outpatient clinics and inpatient wards, mainly due to upper gastrointestinal discomfort. Hp infection was determined by histopathology, ^13^C- or ^14^C-urea breath test (UBT). Inclusion criteria included all infected patients with age range from 18 to 70 years without previously Hp eradication treatment prior to enrollment, and verbal consent was obtained from all patients participated in the study.

Exclusion criteria were as follows: taking antiacid medicines such as PPIs or H_2_-receptor blockers in previous 2 weeks; taking bismuth salts, antibiotics, or other medicines that could influence study results in past 4 weeks; severe concomitant diseases such as liver, kidney, or cardiac dysfunction; planning to be or being pregnant, or lactating; people with mental illness or severe neurosis affecting correct expression or study; history of allergy to any medicines used in current study; and lost in follow-up.

The criteria for terminating study were as follows: serious side effects that could not be tolerated; exacerbation or serious complications such as perforation and gastric hemorrhage; and other serious diseases that require drug intervention.

### 2.2 Drugs

Drugs used in this study were as follows: FZD was from Yunpeng Pharmaceutical Co. Ltd, Shanxi, China; esomeprazole was from AstraZeneca Pharmaceutical Co. Ltd, Jiangsu, China; AMX was from Zhuhai Federal Pharmaceutical Co. Ltd, Guangdong, China; bismuth potassium citrate was from Furen Pharmaceutical Group Co. Ltd, Henan, China; and CLA was from Yangtze River Pharmaceutical Group Co. Ltd, Jiangsu, China. All drugs were routinely prescribed in hospital pharmacy.

### 2.3 Determination of Hp infection in patients

The status of Hp infection was determined by 1 of the 3 methods: positive in histologic staining by upper GI endoscopic biopsy, positive for ^14^C-UBT (HCBT-01, Headway Biological Technology Co. Ltd, Shenzhen, China) or positive for ^13^C-UBT (HY-IREXC 16 channel, Huayou Mingkang Photoelectric Technology Co. Ltd, Guangzhou, China).

UBT was performed after overnight fasting, a baseline breath sample was obtained by blowing certain basal gas into a container, and a powder capsule containing ^13^C-UBT or ^14^C-UBT was given to patients with 80 to 100 mL water, another breath sample was collected 30 minutes later. The test was considered positive if the differences between baseline sample and 30-minute sample exceeded 4.0 arbitrary units for ^13^C-UBT, and 100 arbitrary units for ^14^C-UBT tests. Post-treatment Hp status was assessed by UBT 4 weeks after finishing the treatment, Hp was considered eradicated if UBT result was negative.

### 2.4 Hp eradication regimens and duration

Patients were randomly allocated into 2 groups. FZD group, including FZD (100 mg, bid), esomeprazole (20 mg, bid), bismuth potassium citrate (220 mg, bid), and AMX (1000 mg, bid) for 14 days; CLA group, same as the above drugs except FZD was replaced by CLA (500 mg, bid) for 14 days. Esomeprazole and bismuth were taken 30 minutes before meal, AMX, CLA, and FZD were taken 30 minutes after meal.

### 2.5 Evaluation of symptom remission after treatment and adverse events

Patient's survey results and clinical data were recorded and analyzed. Remission of clinical symptoms, adverse events, and drugs used were recorded by follow-up or outpatient questionnaire sheet.

The symptom grades, based on the degree of affection on patient's daily life and/or work, were classified as follows^[13]^: Level 0, asymptomatic; Level 1, mild with no affection on daily activity; Level 2: moderate, tolerable but affecting daily activity; Level 3: serious and medications were needed. Therapeutic efficacies of all patients were categorized into 3 groups: improved, symptoms disappear; effective, symptoms reduced at least 1 level; ineffective, symptoms not improved or even aggravate. The total effective rate = 1 – inefficiency.

Adverse events were questioned and recorded during treatment period, including time, frequency, duration, severity and whether the patients need to withdrawal or treatment. The severity of adverse events was classified as: no side effect, mild (no limitation in daily activity), moderate (partial limitation in daily activity), severe (significant limitation in daily activity and withdrawal), serious (disability, need to be hospitalized or intervened to prevent permanent injury or even death).

### 2.6 Cost-effectiveness analysis between FZD and CLA groups

Cost (C) is the total cost of each regimen of drugs. Effectiveness (E) represents therapeutic effect of a regimen. In this study, the eradication rate of Hp was used as an indicator of effectiveness. Cost-effectiveness ratio (C/E) and incremental cost-effectiveness ratio were used to evaluate the cost-effectiveness analysis. Incremental cost-effectiveness ratio (ΔC/ΔE) referred to the ratio of cost difference to effectiveness between FZD-based scheme and CLA-based scheme; which means the cost of each unit effect increased by former scheme.

### 2.7 Statistical analysis

Data were analyzed using SPSS software (Version 24, IBM Corp, New York, NY). Continuous variables were presented as mean ± standard deviation, while categorical variables were presented as percentages or frequencies. The efficacy, frequency of side effects, and relieve rate of clinical symptoms were analyzed using chi-squared test or Fisher exact test. Single factor analysis was used to evaluate factors affecting eradication rate. Based on ITT and PP, data efficacy were assessed, *P* values < .05 were considered statistically significant.

## 3 Results

### 3.1 Patients’ demographic and baseline characteristics

**Figure 1:**
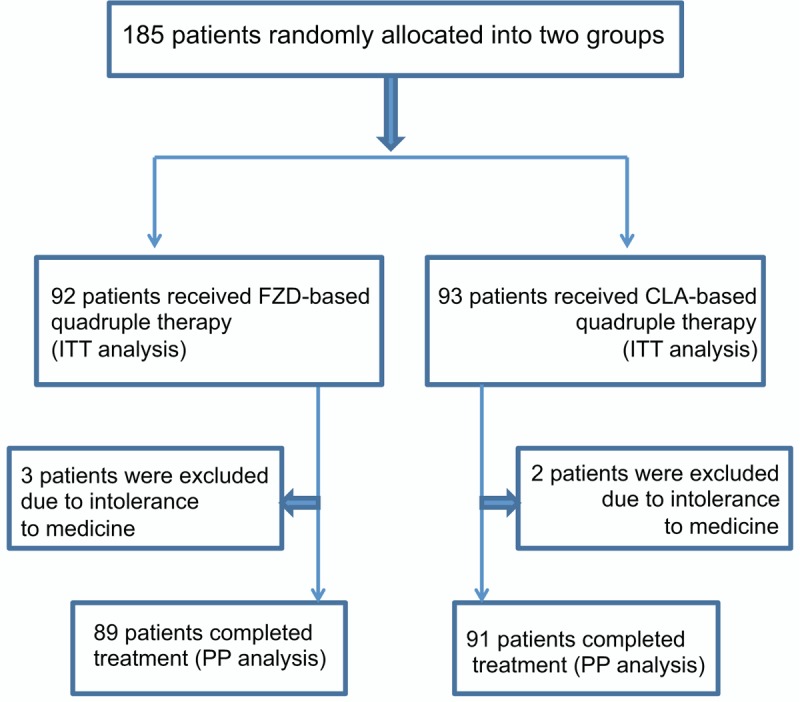
Flow chart of patient enrollment and grouping. CLA = clarithromycin, FZD = furazolidone, ITT = intention-to-treat, PP = per-protocol.

Enrolled patient flowchart was shown in Fig. 1. A total of 185 patients were enrolled and randomly allocated into 2 groups: FZD-based and CLA-based groups. Three patients in FZD group and 2 patients in CLA group discontinued treatment due to adverse effects of therapy. One hundred eighty patients including 89 patients in FZD group and 91 patients in CLA group completed the study with excellent compliance. One hundred forty-six patients completed gastroscopy, of which 31 were peptic ulcers and 115 were chronic; in treatment groups, 2 cases in FZD group, 1 case in CLA group discontinued the study due to intolerance to medicines. Demographic and baseline characteristics of patients were shown in Table 1 and there were no statistical different between 2 groups.

**Table 1:**
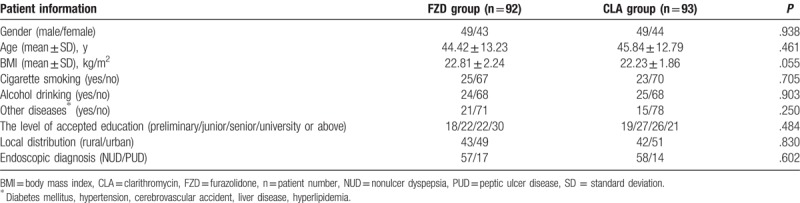
Patient's baseline and demographic data in 2 groups.

### 3.2 Hp eradication rates in FZD and CLA groups

In PP analysis, eradication rates for FZD and CLA groups were 93.26% (83 of 89 cases; 95% CI: 87.9–98.6) and 87.91% (80 of 91 cases; 95% CI: 81.1–94.7), respectively (*P* = .220). In ITT analysis, eradication rates for FZD and CLA groups were 90.22% (83 of 92 cases; 95% CI: 84.0–96.4) and 86.02% (80 of 93 cases; 95% CI: 78.8–93.2), respectively (*P* = .378) (Table 2).

**Table 2:**
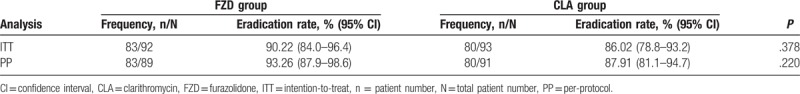
Hp eradication rates in FZD and CLA groups.

### 3.3 Evaluation of symptom remission during Hp-treatment in 2 groups

In FZD group, of the 89 patients who had completed study, the symptoms in 55 cases were improved, in 30 cases were effective, 4 cases were ineffective, and symptom relief rate was 95.51% (85 of 89 cases). In CLA group, of the 91 patients, symptoms in 48 cases were improved, in 38 cases were effective, 5 cases were ineffective, and the total effective rate was 94.51% (86 of 91 cases), no significantly difference was detected between 2 groups (*P* > .05) (Table 3).

**Table 3:**
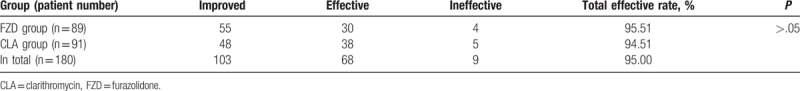
Evaluation of symptom remission in FZD and CLA groups.

### 3.4 Adverse events during Hp-treatment in 2 groups

Table 4 shows the list and proportion of adverse effects, 31 of the 185 patients had adverse events (16.76%). Adverse events occurred in 18 of 92 patients (19.57%) in FZD and 13 of 93 patients (13.98%) in CLA groups, but the rates were not statistically different between the 2 groups (*P* = .309). Majority of adverse effects were mild and moderate, the most common side effects was nausea (9.73%, 18 of 185 cases). The severe side effects were 3.26% (3 out of 92 patients) in FZD group and 2.15% (2 out of 93 patients) in CLA group, and no significant difference was noted between the 2 groups (*P* > .05), these patients discontinued medication; no serious adverse effects were reported.

**Table 4:**
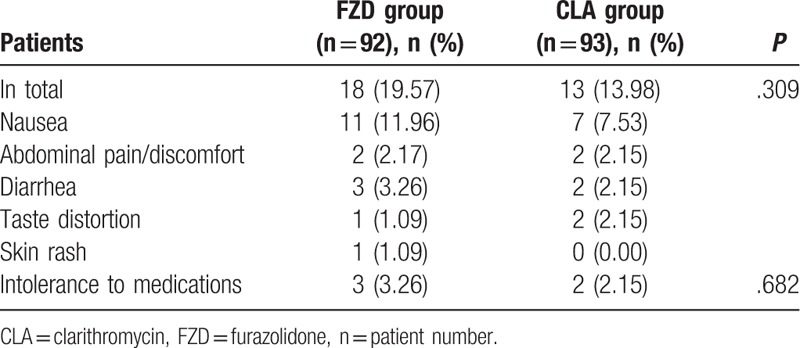
Analysis of adverse events in FZD and CLA groups.

### 3.5 Cost-effectiveness ratio of FZD and CLA groups

Total drug cost of a single treatment course in FZD and CLA treatment group were $70.05 and $89.43, respectively, the cost in FZD group is 20.12% cheaper than that of CLA group. Cost-effectiveness ratios in FZD and CLA groups were 0.75 and 1.02, respectively, and incremental cost-effectiveness ratio was −3.62 (Table 5).

**Table 5:**
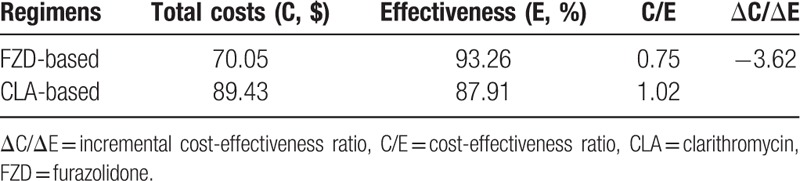
Cost-effectiveness comparison between FZD and CLA regimens.

### 3.6 Factors affecting Hp eradication rate in FZD and CLA groups

The patient's gender, age, body mass index, status of cigarette smoking, level of alcohol drinking, educational level, and urban–rural distribution showed no effects on Hp eradication rates in both groups (*P* > .05); different type of diseases such as diabetes mellitus, hypertension, cerebrovascular accident, liver disease, and hyperlipidemia also do not affect Hp eradication rates in both groups (*P* > .05) (Table 6).

**Table 6:**
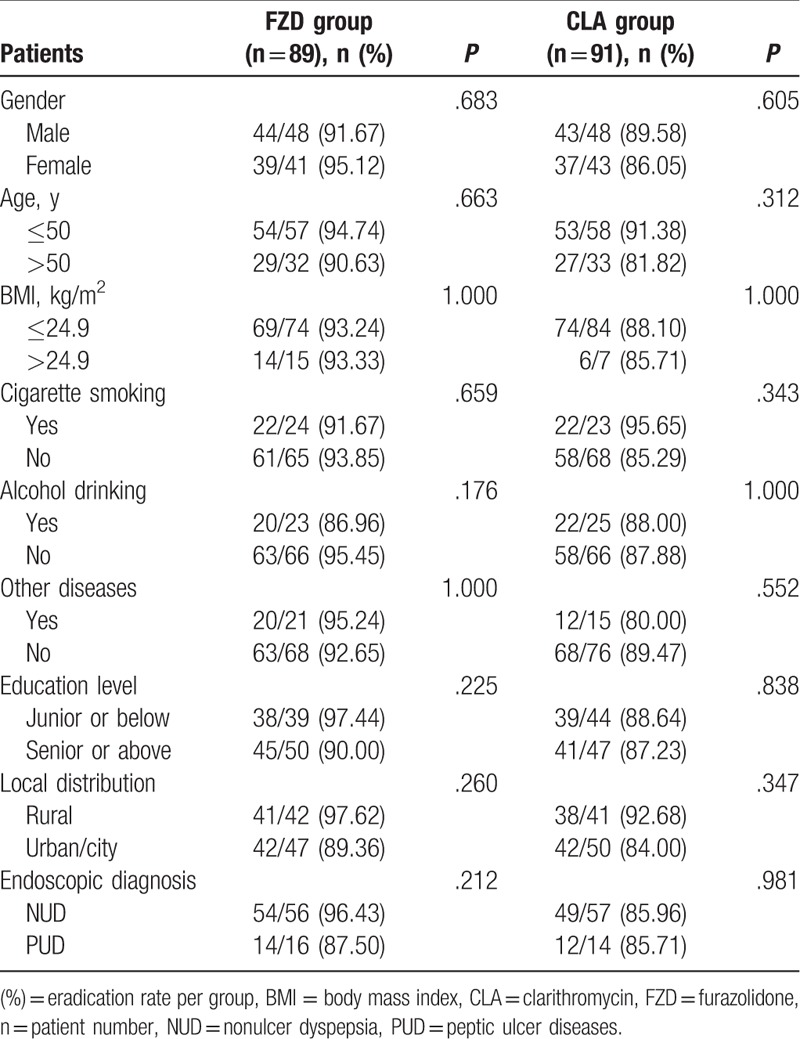
Analysis of factors influencing Hp eradication efficacy.

## 4 Discussion

An optimal anti-Hp regimen is defined as one that reliably produces cure rate of 90% or greater for treatment, and an acceptable therapy as 85% to 89% cure rate.^[14]^ CLA is one of the most commonly used drug in eradicating Hp worldwide with variable eradication rates; however, increasing Hp resistant to CLA has caused concerns for its efficacy during clinical practice. We therefore compare it with FZD, a drug that has fewer drug-resistance incidences, to assess their cost and efficacy during Hp eradication.

Multiple early trails in various doses and regimens such as low dose and sequential therapy have shown that FZD is tolerable and has good Hp eradication rate, and it may replace CLA in various strategies.^[8–11,15]^ Studies in 2001 compared Hp eradication rates with quadruple regimen containing FZD and CLA combined with bismuth, and found no significant difference between 2 groups; Hp eradication rates in FZD and CLA group were 84%, 85% for ITT analysis, respectively, and 90% for both in PP analysis.^[11]^ Our study also shows that FZD-containing regimen achieves satisfactory eradication rate 90.22% in ITT analysis (95% CI: 84.0–96.4) and 93.26% in PP analysis (95% CI: 87.9–98.6). But these results are differ from others who showed that daily 100 mg bid FZD regimen achieved relatively poor results with PP ranging from 56% to 85.7% in Iran (weighted mean ITT was 67%),^[16]^ the difference may be related to geographic distribution, culture, population variations, or patient compliance.

Similarly, the eradication rates of CLA-containing regimens also vary in different regions in China. In 2010, a study from Shanghai evaluated Hp eradication rate using 14-day CLA, PPI, bismuth, AMX quadruple therapy, and noted this regimen achieved eradication rates at 93.7% (95% CI: 88.3–99.0) in ITT analysis and 97.4% (95% CI: 93.8–100) in PP analysis.^[17]^ Another randomized, open-label trail in 2013 investigated CLA-based quadruple 10-day regimen in different regions, and noted the eradication rates in CLA low-resistance area in Shanghai were 81.3% in ITT analysis and 89.7% in PP analysis, compared with CLA-high resistance area in Xi’an with eradication rates only at 50.0% in ITT analysis and 53.6% in PP analysis, respectively,^[9]^ the results suggest that there are geographic variations in CLA-containing regimen. Our study find Hp eradication rates in CLA-based regimen is 86.02% (95% CI: 78.8–93.2) in ITT analysis, and 87.91% (95% CI: 81.1–94.7) in PP analysis. This indicates that Hp has relatively low drug resistance to CLA and AMX combination in this region.

FZD is effective against both gram-negative and gram-positive bacteria, its antibacteria mechanisms are similar to nitroimidazole (such as metronidazole), which mainly interferes with the function of a variety of reductase through 5-nitro group oxidation reduction, thus inhibiting bacteria energy metabolism and exerting antibacterial effect. However, FZD-resistance mechanism is different from metronidazole and does not produce cross resistance.^[18,19]^ The major limitation for widespread use of FZD appears because of its side effects related to digestive tract, such as nausea, anorexia, dizziness, abdomen discomfort etc., which are due to monoamine oxidase inhibitory effects of this drug.^[20]^ Additionally, doses and duration of FZD also affect the incidence of side effects.^[16]^ A study compare the incidence of adverse reactions with 14-day regimens containing FZD has found that as the dose increases from 100 to 200 mg (twice a day), the severe or intolerant side effects leading to the discontinuation of treatment increased from 6% to 20%, including dizziness, abdominal pain, and anorexia.^[10]^

Others note that with higher daily dose of FZD (>200 mg/d), the incidences of some adverse effect such as fever and anorexia were increased over control group, but overall incidences of total side effect and severe side effect did not increase.^[8]^ In our study, at 100 mg bid daily doses, the total incidence of adverse reactions in FZD group is 19.57% (18 of 92 cases), which is not significantly different from CLA group (13.98%, 13 of 93 cases), intolerant side effects occurred only in 3 patients in FZD group and 2 patients in CLA group, and their symptoms disappeared rapidly after withdrawal, no serious adverse consequences were observed. Therefore, low-dose FZD is considered safe and tolerable for use in Hp eradication in present observation.

Our original thought was that CLA regimen would result in much lower eradication rate than that of FZD group, as recent reports have indicated that Hp drug-resistant rates to CLA increased from 14.8% in year 2000 to 52.6% in year 2014 in Beijing, and other studies have reported CLA drug-resistant rate at 22.1% in different regions,^[21,22]^ therefore regimens that containing CLA are assumed to have reduced Hp eradication rates and more treatment failures. However, the results indicate that CLA–AMX regimen still possesses around 85% eradication rate, although we did not perform Hp culture to identify CLA-resistant Hp strains, it appears the second antibiotic, AMX, might be effective regardless of CLA-resistant status. FZD regimen does result in slightly higher eradication rate when compared with CLA group, but the difference is not obvious, the reason is probably due to our patient number are not large enough, future larger number clinical trials should be helpful to address these issues.

The factors that lead to Hp eradication failure include several aspects: antibiotic resistance, bacterial virulence factors, drug pharmacodynamics and pharmacokinetic effects, drug interactions, and poor patient compliance.^[23]^ Among host-related factors, smoking is reported to be associated with Hp eradication failure,^[24,25]^ Itskoviz et al^[25]^ have assessed the effect of smoking on Hp eradication after controlling socio-demographic confounding factors and find that smoking significantly increase the likelihood of first-line Hp treatment failure (OR = 1.15, 95% CI: 1.10–1.20). It has also been reported that females are more likely to fail for eradication (OR = 1.20, 95% CI: 1.14–1.25). However, these claims are not the case in current observation, probably due to patient population and living styles are very different. Further studies are required to understand these discrepancies by conducting large sample size, randomized studies.

This study has some limitations; first, it is a single-center study with relatively small sample size, and majority of patients living around Zhengzhou area. However, as a pilot experiment, the results could be helpful for future investigations involving large sample size, multicenter trails. Second, we did not subgroup our chronic gastritis patients into nonatrophy, atrophy, and intestinal metaplasia groups, which are other potential factors that might affect Hp eradication, as gastroscopy and tissue biopsy were not performed in all patients, this may result in subtle difference in Hp eradication rate which deserve further examination.

In conclusion, despite increasing CLA drug-resistance rate, the present study demonstrates that both FZD-based and CLA-based bismuth quadruple 14-day therapies are effective regimens for Hp eradication; both are safe, tolerable, and have good patient compliance. As FZD-based regimen is more cost-effective, it is therefore recommended as a first-line treatment regimen for clinical practice.

## Acknowledgments

The authors are grateful to the staffs of Department of Gastroenterology and Hepatology, People's Hospital of Zhengzhou University for their valuable assistant in the study. This study was approved by Ethics Committee of People's Hospital of Zhengzhou University, Zhengzhou, China.

## Author contributions

Song-Ze Ding, Jian Li, Yan-Rui Zhang, Yu-Wei Zhang, and Xue-Mei Wang designed the research; Dong-Min Yi, Shuai-Heng Chao, Tao-Tao Yang, Ying-Lei Zhou, Hai-Hui Zhang, Ling Lan, and Ya-Xin Li collected the clinical data; Dong-Min Yi analyzed the data; Dong-Min Yi and Song-Ze Ding wrote the paper; Song-Ze Ding revised the article; all authors approved the final version of manuscript.

**Conceptualization:** Yu-Wei Zhang, Xue-Mei Wang, Yan-Rui Zhang, Jian Li, Song-Ze Ding.

**Data curation:** Tao-Tao Yang, Shuai-Heng Chao, Ya-Xin Li, Ying-Lei Zhou, Hai-Hui Zhang, Ling Lan, Song-Ze Ding.

**Formal analysis:** Dong-Min Yi, Ying-Lei Zhou, Hai-Hui Zhang, Ling Lan, Yan-Rui Zhang, Jian Li.

**Funding acquisition:** Yu-Wei Zhang, Xue-Mei Wang, Song-Ze Ding.

**Investigation:** Dong-Min Yi, Tao-Tao Yang, Shuai-Heng Chao, Ying-Lei Zhou, Hai-Hui Zhang, Ling Lan, Yan-Rui Zhang, Song-Ze Ding.

**Methodology:** Shuai-Heng Chao, Ying-Lei Zhou, Hai-Hui Zhang, Ling Lan, Song-Ze Ding.

**Project administration:** Ya-Xin Li, Yu-Wei Zhang, Xue-Mei Wang.

**Resources:** Yu-Wei Zhang, Jian Li, Song-Ze Ding.

**Supervision:** Xue-Mei Wang, Yan-Rui Zhang, Jian Li, Song-Ze Ding.

**Validation:** Yan-Rui Zhang, Jian Li, Song-Ze Ding.

**Writing – original draft:** Dong-Min Yi, Song-Ze Ding.

**Writing – review & editing:** Song-Ze Ding.

Song-Ze Ding orcid: 0000-0002-4589-6942.
